# Nebulized heparin is associated with fewer days of mechanical ventilation in critically ill patients: a randomized controlled trial

**DOI:** 10.1186/cc9286

**Published:** 2010-10-11

**Authors:** Barry Dixon, Marcus J Schultz, Roger Smith, James B Fink, John D Santamaria, Duncan J Campbell

**Affiliations:** 1 Department of Intensive Care, St. Vincent's Hospital, 41 Victoria Parade, Fitzroy, Melbourne, Victoria, 3065, Australia; 2 Department of Intensive Care Medicine & Laboratory of Experimental Intensive Care and Anesthesiology, The Academic Medical Center, Meibergdreef 9, 1105 AZ, Amsterdam, The Netherlands; 3 Division of Respiratory Therapy, School of Health Professions, College of Health and Human Sciences, Georgia State University, 424 One Park Place South, Atlanta, GA 30302-4019, USA; 4 St. Vincent's Institute of Medical Research, 9 Princes Street, Fitzroy, Melbourne, Victoria, 3065, Australia; 5 Department of Medicine, The University of Melbourne, Clinical Sciences Building, 29 Regent Street, Fitzroy, Melbourne, Victoria, 3065, Australia

## Abstract

**Introduction:**

Prolonged mechanical ventilation has the potential to aggravate or initiate pulmonary inflammation and cause lung damage through fibrin deposition. Heparin may reduce pulmonary inflammation and fibrin deposition. We therefore assessed whether nebulized heparin improved lung function in patients expected to require prolonged mechanical ventilation.

**Methods:**

Fifty patients expected to require mechanical ventilation for more than 48 hours were enrolled in a double-blind randomized placebo-controlled trial of nebulized heparin (25,000 U) or placebo (normal saline) 4 or 6 hourly, depending on patient height. The study drug was continued while the patient remained ventilated to a maximum of 14 days from randomization.

**Results:**

Nebulized heparin was not associated with a significant improvement in the primary end-point, the average daily partial pressure of oxygen to inspired fraction of oxygen ratio while mechanically ventilated, but was associated with improvement in the secondary end-point, ventilator-free days amongst survivors at day 28 (22.6 ± 4.0 versus 18.0 ± 7.1, treatment difference 4.6 days, 95% CI 0.9 to 8.3, *P *= 0.02). Heparin administration was not associated with any increase in adverse events.

**Conclusions:**

Nebulized heparin was associated with fewer days of mechanical ventilation in critically ill patients expected to require prolonged mechanical ventilation. Further trials are required to confirm these findings.

**Trial registration:**

The Australian Clinical Trials Registry (ACTR-12608000121369).

## Introduction

Each year in the US, around 500,000 patients require mechanical ventilation for more than 48 hours [1]. These patients are at high risk of developing lung damage related to inflammatory mechanisms [2]. Acute lung injury (ALI), one manifestation of inflammatory mediated lung damage, is present at the onset of prolonged mechanical ventilation in 18% of patients and subsequently develops in a further 26% of patients [2]. The inflammatory triggers of lung damage include pneumonia, sepsis, aspiration, and trauma [1]. Mechanical ventilation may also damage the lungs through ventilation-induced lung injury and ventilator-associated pneumonia [3-7].

An important inflammatory mechanism of lung damage is fibrin deposition in the pulmonary microcirculation and in the alveolar sacs (hyaline membrane formation). This impairs both alveolar perfusion and ventilation [8-12]. Clinical and experimental models have demonstrated that heparin or other anti-coagulants reduce fibrin deposition in the lungs and improve clinical outcomes [13-17]. Heparin has other actions, including reduced pulmonary edema, reduced leukocyte activation, and inhibition of adhesion of bacteria and viruses to respiratory surfaces, that may also be beneficial [18-22]. Evidence from large, multi-center, clinical studies in patients with severe sepsis also suggests that heparin may improve important clinical outcomes. *Post hoc *analysis of three interventional studies found that prophylactic subcutaneous heparin administration was associated with reduced mortality (32% versus 42%, *P *= 0.0001) [23-26]. Furthermore, a subsequent prospective randomized study of subcutaneous heparin in patients treated with activated protein C for severe sepsis also found a trend of lower mortality (28% versus 32%, *P *= 0.08) [27].

Nebulization of heparin may offer benefits over systemic administration because nebulization enhances delivery to the bronchial tree and the alveolar sacs and reduces the potential for systemic bleeding associated with intravenous administration. Furthermore, nebulized heparin has been shown to reduce levels of coagulation activation in the lungs both in animal studies and in patients with ALI [28-31]. In this study, we therefore assessed whether nebulized heparin improved lung function in patients expected to require prolonged mechanical ventilation.

## Materials and methods

### Patients

The study, Can Heparin Reduce Lung Injury (CHARLI), took place between July 2008 and November 2009. We studied patients admitted to the intensive care unit (ICU) of St. Vincent's Hospital (Melbourne, Australia), a tertiary-level university-affiliated hospital. The St. Vincent's Hospital Human Research Ethics Committee approved the study. Informed consent was obtained from the patient, next of kin, or appropriate surrogate before participation in the study. The study was registered with the Australian Clinical Trials Registry (ACTR-12608000121369).

Patients were included if, owing to primary respiratory failure or other indications, they were expected to require invasive mechanical ventilation for more than 48 hours. They were excluded if they received mechanical ventilation for more than 24 hours prior to enrollment, required mechanical ventilation for more than 48 hours in a previous admission to the ICU during the current hospital admission, or received any of the following at the time of screening: high-frequency ventilation, extracorporeal membrane oxygenation, nitric oxide (NO), renal replacement therapy, therapeutic doses of heparin or low-molecular-weight heparin, warfarin, drotrecogin alpha activated, or protamine. Also, they were excluded if the physician was not committed to full supports or they had a body mass index of 40 kg/m^2 ^or greater, allergy to heparin (including any history of heparin-induced thrombocytopenia), a pulmonary hemorrhage in the previous 3 months, uncontrolled bleeding or a significant bleeding disorder, an intracranial hemorrhage in the past 12 months (a clipped subarachnoid aneurysm was acceptable), or an epidural catheter in place or likely to be placed in the next 48 hours or were younger than 18 years old.

### Study design

CHARLI was a double-blind, randomized, placebo-controlled trial. Block randomization was performed in random blocks of two to eight. Randomization was stratified by the presence of ALI at enrollment [32]. Allocations were concealed in opaque, sequentially numbered, sealed envelopes.

### Study medication

Heparin and placebo were presented in identical 5-mL plastic ampules: heparin sodium (porcine mucous) 25,000 U/5 mL (Pfizer, West Ryde, Australia) and placebo (0.9% sodium chloride; Pfizer). Patients were administered 5 mL of study medication every 4 hours or, if they were less than 165 cm in height, every 6 hours. The dose was based on data from an earlier study of patients with ALI [28,29]. Study medication was continued with a dose regimen of every 6 hours if therapeutic anti-coagulation was commenced. No dose adjustment was made for heparin administration for deep venous thrombosis prophylaxis. The study medication was continued while the patient remained ventilated and was given for a maximum of 14 days from randomization. The study medication was reduced or withheld at the physicians' discretion if any of the following occurred: excessive blood staining of the sputum, other significant bleeding, a planned surgical procedure, or an excessively elevated activated partial thromboplastin time (APTT).

### Nebulization

Heparin or placebo was nebulized via an Aeroneb Pro nebulizer (Aerogen Ltd., Galway, Ireland) for 30 minutes. The nebulizer generates droplets with a mass median aerodynamic diameter of 2.1 μm. The nebulizer was placed in the inspiratory limb just before the Y-piece. An active humidification system was used, and humidification was continued during nebulization (Fisher & Paykel Healthcare Ltd., Auckland, New Zealand). A filter was placed in the expiratory limb of the circuit to prevent the nebulized study drug from damaging the expiratory valve of the ventilator (BB50TE; Pall Corporation, Port Washington, NY, USA, or RT019; Fisher & Paykel Healthcare Ltd.). This filter was changed at least daily.

### Ventilation

A pressure-controlled mode of mechanical ventilation was used. The target tidal volume was set at not more than 8 mL/kg of predicted body weight; this was routine practice at the time of the study. The predicted body weight was calculated as previously described [7]. Weaning was undertaken with a spontaneous pressure support mode. The level of pressure support was adjusted in order to maintain the target tidal volume. NO was considered if hypoxemia was present despite an inspired fraction of oxygen (FiO_2_) of more than 60% and a positive end-expiratory pressure (PEEP) of more than 10 cm H_2_O or if pulmonary hypertension with hemodyamic instability was present. Patients were considered suitable for extubation if they were cooperative and hemodynamically stable with an oxygen saturation of at least 95% while ventilated on pressure support of not more than 10 cm H_2_O, PEEP of not more than 5 cm H_2_O, and FiO_2 _of not more than 50%. Patients who were not suitable for extubation after 4 days of mechanical ventilation and who had not demonstrated clinical improvement were considered for tracheostomy. Routine tracheostomy insertion was undertaken, using a percutaneous technique, by the treating intensive care physician.

### Outcomes

The primary outcome was the average daily ratio of partial pressure of oxygen to FiO_2 _(PaO_2_/FiO_2_) while the patient remained ventilated for a maximum of 14 days from randomization. Secondary outcomes included ventilator-free days among surviving patients at day 28, the development of ALI following enrollment, tracheostomy rate, days free of vasopressor and acute renal failure among survivors at day 28, lengths of stay in ICU and hospital, and mortality at days 28 and 60.

### Anti-coagulant effects of heparin

Daily APPT levels were recorded to assess the systemic effects of nebulized heparin. Pulmonary lavage markers of coagulation activation (thrombin-antithrombin complex [TAT] and D-dimer) were also measured.

### Pulmonary lavage

Levels in pulmonary lavage fluid of inflammatory mediators (tumor necrosis factor-alpha [TNF-α], interleukin [IL]-6, and IL-8), of lung damage (surfactant protein-D [SP-D], Clara cell protein-16 [CC-16], and receptor for advanced glycation end-products [RAGE]), and TAT and D-dimers were measured at baseline and on study days 1, 2, 4, 8, and 14 if the patient remained mechanically ventilated and sedated. In addition, cultures were undertaken on the pulmonary lavage samples.

### Data collection

The PaO_2_/FiO_2 _ratio and oxygenation index (FiO_2 _multiplied by the mean airway pressure divided by the PaO_2_) were measured each day at 4 a.m. No changes in the ventilator settings or the patient's position were permitted for the 10 minutes before this measurement. A non-bronchoscopic pulmonary lavage was performed using a suction catheter, as previously described [33]. The recovered fluid was centrifuged at 1,500*g *for 10 minutes at 4°C. The supernatant was collected and stored at -80°C until measurements were performed. Levels of TAT, D-dimer, TNF, IL-6, IL-8, RAGE, CC-16, and SP-D were assessed by enzyme-linked immunosorbent assay as previously described [30,34].

Demographic data were collected on study entry, and ventilation parameters, clinical and radiological data, sputum character, medication usage, and adverse events, including blood-stained sputum or frank blood in sputum and red cell transfusions, were recorded daily while the patient remained mechanically ventilated. Ventilator-free days was defined as the number of days patients were breathing without mechanical ventilation during the first 28 days. Development of ALI was defined using the consensus criteria [32]. Vasopressor usage was defined by the administration of any of the following: dopamine, dobutamine, norepinephrine, or epinephrine. Renal failure was defined as a serum creatinine of greater than 300 μmol/L or urine output of less than 500 mL per day or renal replacement therapy for acute renal impairment. Respiratory failure was defined as the acute requirement for mechanical ventilation primarily due to ALI, pneumonia, influenza, aspiration, exacerbation of chronic obstructive airway disease, or other acute lung disorder.

### Statistical analysis

On the basis of previous data from a trial of intravenous heparin to limit lung injury in patients undergoing cardiac surgery, the study was powered to demonstrate an improvement in the average daily PaO_2_/FiO_2 _ratio from 250 to 300 mm Hg over the period of mechanical ventilation, assuming a standard deviation (SD) of 50, alpha = 0.05, and power = 0.8 [35]. Data were analyzed on an intention-to-treat basis. Data are expressed as mean ± standard error of the mean or SD, or as median with interquartile range, and were compared using Student *t *test or the median test where appropriate. Categorical variables were compared using chi-square tests or Fisher exact tests where appropriate. The rate of freedom from mechanical ventilation was analyzed according to the Kaplan-Meier method and the results were compared with the log-rank test. All reported *P *values were two-sided. A *P *value of 0.05 or less was considered to indicate statistical significance. Analyses were conducted with JMP software (SAS Institute Inc., Cary, NC, USA).

## Results

### Enrollment data

**Figure 1:**
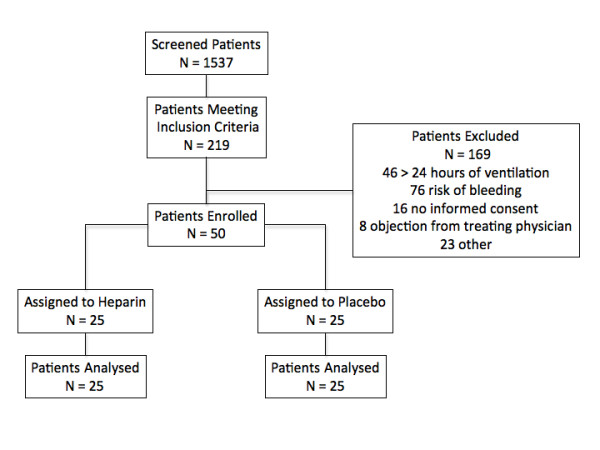
**Enrollment and outcomes**.

**Figure 2:**
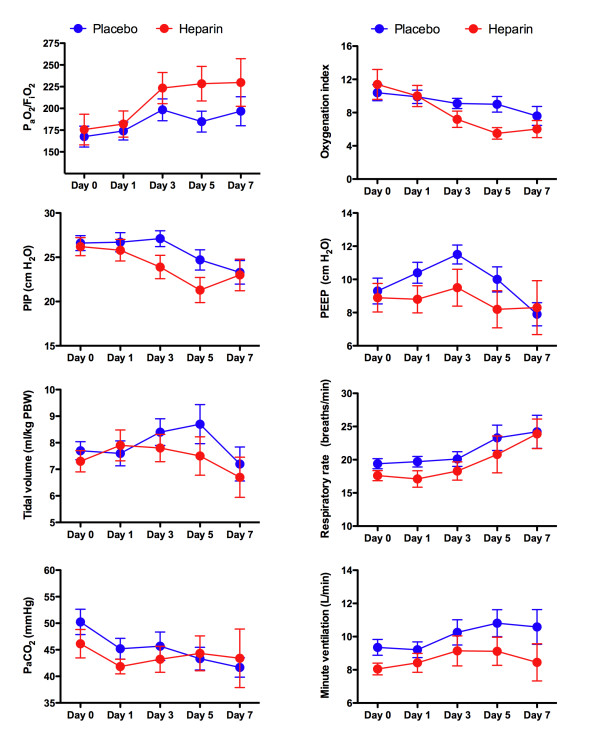
**Respiratory values over the first 7 days of the study**. There were no significant differences between groups in the average daily levels of the ratio of partial pressure of arterial oxygen to fraction of inspired oxygen (PaO_2_/FiO_2_), predicted body weight (PBW), peak inspiratory pressure (PIP), positive end-expiratory pressure (PEEP), partial pressure of arterial carbon dioxide (PaCO_2_), or minute ventilation over the course of the study period (days on which the patient remained mechanical ventilation to a maximum of 14 days from randomization). The numbers of patients who were mechanically ventilated are as follows: day 0 placebo (25) and heparin (25), day 1 placebo (24) and heparin (25), day 3 placebo (21) and heparin (15), day 5 placebo (19) and heparin (11), and day 7 placebo (13) and heparin (7). Graphs represent mean ± standard error of the mean.

Screening and enrollments are shown in Figure 1. Twenty-five patients were randomly assigned to nebulized heparin, and 25 were randomly assigned to placebo. All patients received the allocated treatment and were included in the final analysis. The baseline characteristics of the two groups, including the APACHE II (Acute Physiology and Chronic Health Evaluation II) score and the proportion of patients with respiratory failure or ALI and APTT levels, were similar (Table 1). In addition, the respiratory physiological variables at enrollment were similar between groups (Figure 2). A trend of increased positive respiratory cultures in the placebo group was present at baseline (11/25 [44%] versus 5/25 [20%], relative risk 0.5, 95% confidence interval [CI] 0.2 to 1.2, *P *= 0.07) (Table 2).

**Table 1:** Baseline characteristics

Baseline characteristics	Placebo *N *= 25	Heparin *N *= 25
Age in years, mean ± SD	55.5 ± 17.0	56.0 ± 16.5
Males, number (percentage)	14 (56)	18 (72)
APACHE II score, mean ± SD	19.4 ± 7.2	20.2 ± 6.1
Respiratory failure^a^, number (percentage)	17 (68)	14 (56)
Acute lung injury, number (percentage)	4 (16)	4 (16)
Aspiration, number (percentage)	2 (8)	2 (8)
Vasopressor use, number (percentage)	16 (64)	13 (52)
APTT, seconds	41.1	38.0
Primary diagnosis, number (percentage)		
Community-acquired pneumonia	9 (36)	7 (28)
Hospital-acquired pneumonia	3 (12)	2 (8)
H1N1 influenza	1 (4)	2 (8)
Chronic obstructive airway disease	1 (4)	1 (4)
Post cardiac arrest	1 (4)	2 (8)
Cardiac failure	2 (8)	1 (4)
Meningitis	1 (4)	0
Uncontrolled seizures	0	1 (4)
Subarachnoid hemorrhage	1 (4)	1 (4)
Cardiac surgery	3 (12)	4 (16)
Thymectomy	0	1 (4)
perforated duodenal ulcer	1 (4)	0
Cervical fracture	0	1 (4)
Drug intoxication	2 (8)	2 (8)
Admission source, number (percentage)		
Emergency department	13 (52)	9 (36)
Operation theater	4 (16)	5 (20)
Hospital ward	6 (24)	8 (32)
Other hospital	2 (8)	3 (12)

^a^Denotes acute requirement for mechanical ventilation primarily due to acute lung injury, pneumonia, influenza, aspiration, exacerbation of chronic obstructive airway disease, or other acute lung disorder. APACHE II, Acute Physiology and Chronic Health Evaluation II; APPT, activated partial thromboplastin time; SD, standard deviation.

**Table 2:** Pulmonary lavage values

	Baseline		Day 1		Day 2		Day 4	
				
	Placebo *N *= 25	Heparin *N *= 25	Placebo *N *= 24	Heparin *N *= 24	Placebo *N *= 22	Heparin *N *= 20	Placebo *N *= 18	Heparin *N *= 14
TAT, μg/L	13.9 (6.2-37.9)	8.7 (3.5-30.0)	7.8 (4.9-20.7)	8.1 (3.5-17.3)	9.3 (3.9-15.7)	7.3 (3.1-20.3)	7.9 (4.3-11.0)	7.2 (2.6-18.4)
D-dimer, mg/L	3.1 ± 6.3	2.4 ± 7.5	5.4 ± 11.3	2.8 ± 7.8	1.3 ± 2.0	0.67 ± 1.1	0.67 ± 1.1	0.52 ± 0.54
IL-6, pg/mL	235 (44-1,699)	187 (34-961)	173 (71-547)	182 (58-589)	132 (36-497)	105 (27-478)	90 (34-313)	60 (23-227)
IL-8, pg/mL	546 (219-1,383)	559 (198-11,949)	667 (255-2,633)	1,396 (333-6,009)	874 (381-1,901)	1328 (369-2,895)	691 (234-1,307)	1,035 (246-1,812)
TNFα, pg/mL	11.8 ± 19.5	9.3 ± 12.7	7.3 ± 10.2	4.9 ± 7.3	5.9 ± 8.9	6.8 ± 13.3	7.9 ± 9.9	6.4 ± 7.9
SP-D, ng/mL	162.6 (44.7-602.3)	309.7 (51.7-805.9)	305.1 (65.3-662.1)	210.5 (36.0-901.5)	153.8 (82.7-823.2)	119.2 (78.4-368.1)	136.1 (85.5-309.4)	176.6 (43.8-399.6)
CC-16, ng/mL	180.0 ± 148.6	207.2 ± 137.8	176.8 ± 155.7	172.4 ± 123.7	225.5 ± 148.6	186.5 ± 150.0	229.8 ± 142.6	207.4 ± 237.9
RAGE, pg/mL	2,015 ± 3,607	3,170 ± 5,428	3,717 ± 11,072	3,320 ± 6,472	1,564 ± 3,273	1,975 ± 3,633	563 ± 1,369	936 ± 1,737

Values are presented as median and interquartile range (there were no statistical differences between groups, and comparisons were undertaken at baseline and study days 1, 2, and 4) or as mean ± standard deviation. CC-16, Clara cell protein-16; IL, interleukin; RAGE, receptor for advanced glycation end-products; SP-D, surfactant protein-D; TAT, thrombin-antithrombin complex; TNFα, tumor necrosis factor-alpha.

### Clinical outcomes

The primary end-point, the average daily PaO_2_/FiO_2 _ratio while ventilated, was similar in the heparin and placebo groups (194.2 ± 62.8 versus 187 ± 38.6 mm Hg, mean difference 7.2, 95% CI -22.8 to 37.1, *P *= 0.6). Though not statistically significant, the PaO_2_/FiO_2 _ratio levels were higher from day 3 in the heparin group. In addition, NO was used less frequently in the heparin group (0/25 [0%] versus 5/25 [19%], relative risk 0.8, 95% CI 0.7 to 0.97, *P *= 0.05). The PaO_2_/FiO_2 _ratio levels and other respiratory variables over the first 7 days are presented in Figure 2.

**Figure 3:**
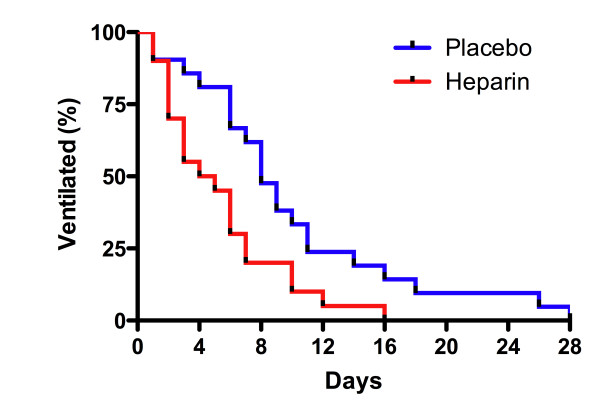
**Rate of freedom from mechanical ventilation**. Over the first 28 days among surviving patients, the rate of freedom from mechanical ventilation was higher in patients administered heparin. Median times of ventilation were 5 days in the heparin group (*n *=  20) and 8 days in the placebo group (*n *=  21) (*P *= 0.01) (log-rank test).

Heparin administration was associated with a higher number of ventilator-free days among survivors at day 28 (22.6 ± 4.0 versus 18.0 ± 7.1, treatment difference 4.6, 95% CI 0.9 to 8.3, *P *= 0.02). Similarly, when the composite method was used to calculate ventilator-free days (patients who died were assigned 0 ventilator-free days), there was a difference between groups (22 [14 to 26] versus 19 [6 to 22], *P *<0.05). The rate of freedom from mechanical ventilation among survivors at day 28 was higher in the heparin group (*P *= 0.01, log-rank test) (Figure 3). The number of tracheostomies tended to be lower in the heparin group (7/25 [28%] versus 12/25 [48%], relative risk 0.66, 95% CI 0.39 to 1.1, *P *= 0.1). A tracheostomy, if required, was undertaken an average of 5 ± 3 days after enrollment. There was also a trend of reduced development of ALI following enrollment in the heparin group (0/21 [0%] versus 4/21 [19%], relative risk 0.84, 95% CI 0.7 to 1.0, *P *= 0.1).

The number of vasopressor-free days among survivors at day 28 (24.7 ± 3.2 versus 22.0 ± 7.0 days, difference -2.7, 95% CI -6.2 to 0.8, *P *= 0.12) and the number of renal failure-free days among survivors at day 28 (28 [28 to 28] versus 28 [26.5 to 28] days, *P *= 0.09) were higher in the heparin group but did not reach statistical significance. The ICU and hospital lengths of stay (9.4 ± 7.4 versus 14.0 ± 13.1 days, difference -4.7, 95% CI -11.4 to 2.1, *P *= 0.2, and (24 [15 to 36.5] versus 27 [16.0 to 52.5] days, *P *= 0.4) and mortality at days 28 and 60 (20% versus 16%, *P *= 0.7, and 28% versus 20%, *P *= 0.5) were similar in the two groups.

### Anti-coagulant effects of heparin

The maximum increase in the APTT from baseline levels over the study period was higher in the heparin group (4 [1 to 13] versus 0 [-1 to 4] seconds, *P *= 0.02). This analysis excluded patients administered therapeutic heparin. Pulmonary lavage levels of TAT and D-dimer were similar in the two groups at baseline and following enrollment (Table 2).

### Respiratory cultures and pulmonary inflammatory markers

The proportion of patients with new positive respiratory cultures following enrollment was similar in the two groups (Table 3). Levels of IL-6, IL-8, TNF, SD-P, CC-16, and RAGE in pulmonary lavage fluid were also similar in the two groups at baseline and on each day that samples were taken following enrollment (Table 2).

**Table 3:** Respiratory microbiology

	Positive culture at enrollment		*P *value	New positive culture following enrollment		*P *value
				
	Placebo *N *= 25	Heparin *N *= 25		Placebo *N *= 25	Heparin *N *= 25	
Respiratory cultures, number (percentage)						
Pathogen detected	11 (44)	5 (20)	0.07	12 (48)	9 (36)	0.4
Gram-positive	4 (16)	1 (4)	0.4	1 (4)	2 (8)	0.5
Gram-negative	5 (20)	1 (4)	0.2	6 (24)	5 (20)	0.7
Yeast/fungus	3 (12)	1 (4)	0.6	7 (28)	3 (7)	0.2
Legionella pneumonia	1 (4)	0	1	0	0	
H1N1 polymerase chain reaction	1 (4)	2 (8)	1	0	0	

Pulmonary lavage was undertaken at enrollment and on study days 1, 2, 4, 8, and 14 if the patient remained ventilated and sedated.

### Other medication

Therapeutic doses of systemic heparin were administered to 24% of patients in the heparin group and 32% in the placebo group following enrollment. Heparin for deep venous thrombosis prophylaxis was administered to 64% of patients in the heparin group and 48% in the placebo group prior to enrollment and to 84% of patients in both groups following enrollment. Antibiotics, steroids, and other nebulized medications were used to a similar extent in the two groups (Supplement in Additional file 1).

### Safety and tolerability

On average, each patient in the heparin group was administered 22 ± 15 doses and each patient in the placebo group was administered 37 ± 20 doses. The study drugs were well tolerated, with only 6% of scheduled doses withheld in the heparin group and 4% in the placebo group during the study period (Table 4). The percentages of days during the study period patients had blood-stained sputum were similar in the heparin and placebo groups (41 ± 39 versus 31 ± 28). Blood product usage was also similar for the two groups, with 7 patients (28%) transfused over the study period in the heparin group and 10 patients (40%) transfused in the placebo group. No patients had blood loss or transfusion requirements attributable to the study medication. One patient in the heparin group had a marked increase for 3 days in the APTT to more than 150 seconds; this corrected with temporary withdrawal of the study medication.

**Table 4:** Study drug safety and tolerability

	Placebo *N *= 25	Heparin *N *= 25	*P *value
Percentage of scheduled doses withheld	3.8 ± 9.2	5.8 ± 9.5	0.5
Reasons for withholding			
Blood-stained sputum	1 (4)	6 (24)	0.1
Prolonged APTT	1 (4)	3 (12)	0.6
Suspected HIT	0	1 (4)	1
Confirmed HIT	1 (4)	0	1
Invasive procedure	3 (12)	0	0.2
Other	4 (16)	2 (8)	0.7
Percentage of days during study period with			
Blood-stained sputum	31 ± 28	41 ± 39	0.3
Frank blood in sputum	2.5 ± 9	7.0 ± 21	0.3
Purulent sputum^a^	62 ± 25	61 ± 35	1
Red cell transfusion			
Volume transfused per day (mls)	0 (0-57)	0 (0-85)	0.9
Patients transfused	10 (40)	7 (28)	0.4
Abnormal APTT^b ^(> 40 seconds)	5/17 (29)	11/19 (58)	0.08

^a^Denotes sputum reported as yellow, green, dirty, or purulent. ^b^Patients on therapeutic heparin were excluded from this analysis. Values other than *P *values are presented as mean ± standard deviation, as median (interquartile range), or as number (percentage). APPT, activated partial thromboplastin time; HIT, heparin-induced thrombocytopenia.

### Subgroup analysis by Forest plot

**Figure 4:**
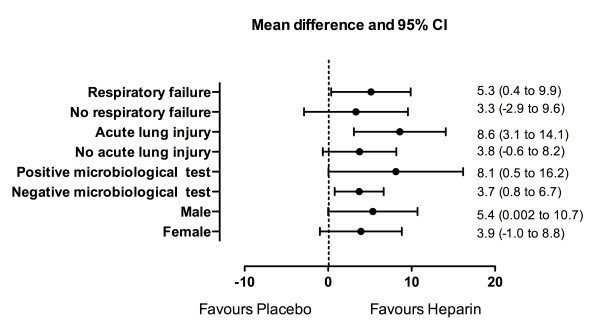
**Forest plot of the treatment effect of nebulized heparin on ventilator-free days among survivors at day 28 by baseline characteristics**. The microbiological tests included culture of pulmonary lavage fluid and polymerase chain reaction for H1NI. CI, confidence interval.

We conducted a *post hoc *subgroup analysis to examine the effects of enrollment characteristics on the ventilator-free days among survivors at day 28. The magnitude of the treatment effect of heparin was similar regardless of whether respiratory failure, ALI, or a positive respiratory microbiological test was present at enrollment (Figure 4).

## Discussion

In this double-blind, randomized, placebo-controlled trial of nebulized heparin in critically ill patients expected to require prolonged mechanical ventilation, we found that nebulized heparin was associated with fewer days of mechanical ventilation. We found no difference between groups in the primary end-point, the average daily PaO_2_/FiO_2 _ratio while ventilated. The more rapid rate of extubation in the heparin group limited the power of the study to demonstrate a difference in this end-point, as the daily PaO_2_/FiO_2 _ratio was measured only if the patient was ventilated. Consequently, it was not possible to continue to collect the PaO_2_/FiO_2 _ratio data from the most improved patients. The daily changes in the PaO_2_/FiO_2 _ratio support this rationale with higher levels of oxygenation over the first 7 days in the heparin group (Figure 2). In addition, the increased use of NO following enrollment in the placebo group (20%) compared with the heparin group (0%) indicates that oxygenation was less problematic in the heparin group.

Whereas we found no statistically significant difference in the PaO_2_/FiO_2 _ratio, we did find a significant improvement in the clinically important end-point of ventilator-free days. One mechanism by which nebulized heparin may improve the potential for successful extubation is through reducing fibrin deposition in the pulmonary microcirculation and the alveolar sacs (hyaline membrane). Recent randomized studies in patients with acute pulmonary inflammation following cardiac surgery demonstrated that intravenous heparin significantly reduced histological and other evidence of pulmonary microvascular thrombosis [13,35]. Animal models of sepsis and ALI have also demonstrated reduced microvascular thrombosis and hyaline membrane formation with both intravenous and nebulized heparin [16,17,36-39]. Fibrin deposition creates a barrier to gas exchange by reducing both alveolar perfusion and ventilation [8-12]. Pulmonary microvascular thrombosis may also cause ischemic damage of alveolar tissue and right heart strain through increased right-ventricle after-load [23,40,41]. Furthermore, fibrin is a proinflammatory mediator that plays an important role in leukocyte recruitment to inflamed tissues, and this may also damage lung tissues [42].

We assessed the anti-coagulant effects of nebulized heparin on the lungs by measuring systemic APTT levels and also markers of coagulation activation in pulmonary lavage fluid. Nebulized heparin was associated with a systemic anti-coagulant effect with a greater increase in APTT levels compared with placebo. The local anti-coagulant effects of nebulized heparin in the alveolar sacs and microcirculation of the lungs therefore would be much greater. Nebulized heparin is cleared slowly from the lungs, and 40% was still present in the lungs 24 hours after nebulization of a single dose [43,44]. It was surprising, therefore, that the pulmonary lavage markers of coagulation activation were not lower in the heparin group. We believe that this finding reflects methodological limitations. In an earlier clinical study of patients with ALI, nebulized heparin did significantly reduce coagulation activation in the lungs [28]. In this study, bronchoscopic methods were used to obtain alveolar lavage fluid, whereas in the present study, non-bronchoscopic methods (a suction catheter placed in the bronchial tree) were used to obtain pulmonary lavage fluid. A recent comparison of the two techniques found that non-bronchoscopic methods did not provide an accurate assessment of alveolar levels of lung inflammatory markers [45]. This methodological limitation may also be a factor in the finding of similar levels of inflammatory mediators (IL-6, IL-8, and TNF) and markers of lung damage (SP-D, CC-16, and RAGE) in the pulmonary lavage fluid of the two groups [46].

Previous studies have suggested that heparin may inhibit growth of bacteria and viruses in the lungs through limiting their adhesion to respiratory surfaces [20-22]. We did not find evidence to support this, and there were similar numbers of new positive bacterial cultures in pulmonary lavage fluid in both groups following enrollment.

### Safety and adverse events

Heparin nebulization was safe and not associated with serious adverse events, even in patients co-administered systemic heparin. The study drugs were well tolerated, and only 6% of scheduled doses were withheld in the heparin group and 4% of scheduled doses were withheld in the placebo group. The percentage of study days with blood-stained sputum was relatively common but was not significantly increased in the heparin group. Blood product usage was also similar, and no blood transfusion was attributable to the study medication. The maximum change in the APTT from baseline levels over the study period was higher in the heparin group. This increase was not associated with any adverse clinical consequences. In one patient, the APTT level exceeded 150 seconds. This corrected by omitting the nebulized heparin for 24 hours. After resuming heparin at a lower dose, the APPT levels remained stable.

### Limitations and strengths

The indications for mechanical ventilation were relatively broad. At enrollment, 62% of patients were ventilated for respiratory failure, whereas the remaining patients were ventilated for other reasons, including cardiac failure and coma. However, the proportion of patients ventilated for respiratory failure or ALI was similar in the two groups. Furthermore, *post hoc *subgroup analysis indicated that the magnitude of the treatment effect was similar regardless of the indication for mechanical ventilation and of whether ALI was present at enrollment. Another potential limitation of the study was that it was undertaken in a single center. This raises questions regarding whether our findings can be applied to other settings. The study, however, had important strengths, including the fact that all patients were exposed to prolonged mechanical ventilation and therefore the associated risks of lung damage. In addition, the double-blind placebo design and randomization of patients limited the potential for bias.

## Conclusions

Nebulized heparin was associated with fewer days of mechanical ventilation in patients expected to require prolonged mechanical ventilation. Further trials are required to confirm these findings.

## Key messages

• Nebulized heparin may reduce the duration of mechanical ventilation in critically ill patients expected to require prolonged mechanical ventilation.

## Abbreviations

ALI: acute lung injury; APTT: activated partial thromboplastin time; CC-16: Clara cell protein-16; CHARLI: Can Heparin Reduce Lung Injury; CI: confidence interval; FiO_2_: inspired fraction of oxygen; ICU: intensive care unit; IL: interleukin; NO: nitric oxide; PaO_2_: partial pressure of oxygen; PEEP: positive end-expiratory pressure; RAGE: receptor for advanced glycation end-products; SD: standard deviation; SP-D: surfactant protein-D; TAT: thrombin-antithrombin complex; TNF: tumor necrosis factor.

## Competing interests

St. Vincent's Hospital has applied for a patent relating to nebulized heparin, and BD and RS could benefit from this application. The other authors declare that they have no competing interests.

## Authors' contributions

BD and RS participated in the study design, data gathering, interpretation, statistical analysis, and writing the first draft and all revisions of the manuscript. MJS participated in the study design, laboratory analysis, interpretation, and writing the first draft and all revisions of the manuscript. JBF participated in the study design. DJC and JDS participated in the study design and revisions of the manuscript. All authors read and approved the final manuscript.

## Acknowledgements

We wish to thank Karl Askew and Joe Walters, who greatly assisted in the randomization of patients and study drug preparation. This work was supported by a grant from the Australian Intensive Care Foundation. The funding body played no role in study design; in collection, analysis, or interpretation of data; in writing of the manuscript; or in the decision to submit the manuscript for publication.

## Supplementary Material

Additional file 1 **Supplement**. A table of medication. Click here for file
