# Supplementary material for: Nebulized heparin is associated with fewer days of mechanical ventilation in critically ill patients: a randomized controlled trial
Source: Crit Care. 2010 Oct 11;14(5):R180. doi: 10.1186/cc9286 (PMC3219284; doi:10.1186/cc9286)
Supplement: Additional file 1 — Supplement. A table of medication. [file cc9286-S1.DOC]

Table 1. Medication

|  | Baseline | | P  value | Study period* | | P  value |
| --- | --- | --- | --- | --- | --- | --- |
|  | Placebo  N=25 | Heparin  N=25 |  | Placebo  N=25 | Heparin  N=25 |  |
| Medication |  |  |  |  |  |  |
| Vancomycin, n (%) | 6 (24) | 8 (32) | 0.4 | 14 (56) | 13 (52) | 0.8 |
| Ceftriaxone, n (%) | 16 (64) | 17 (68) | 0.8 | 17 (68) | 18 (72) | 0.8 |
| Meropenem, n (%) | 1 (4) | 3 (12) | 0.6 | 8 (32) | 9 (36) | 0.8 |
| Timentin, n (%) | 4 (16) | 1 (4) | 0.3 | 9 (36) | 6 (24) | 0.4 |
| Azithromycin, n (%) | 12 (48) | 7 (28) | 0.1 | 13 (52) | 11 (44) | 0.6 |
| Other antibiotic, n (%) | 12 (48) | 9 (36) | 0.4 | 17 (68) | 11 (44) | 0.09 |
| Steroids, n (%) | 7 (28) | 7 (28) | 1 | 13 (52) | 10 (40) | 0.4 |
| Nebulized steroids, n (%) | 1 (4) | 2 (8) | 1 | 4 (16) | 6 (24) | 0.7 |
| Nebulized bronchodilators, n (%) | 9 (36) | 4 (16) | 0.1 | 14 (56) | 11 (44) | 0.4 |
| Prophylactic heparin, n (%) | 16 (64) | 12 (48) | 0.4 | 21 (84) | 21 (84) | 1 |
| Therapeutic heparin, n (%) |  |  |  | 8 (32) | 6 (24) | 0.5 |
| Nitric oxide, n (%) |  |  |  | 5 (20) | 0 | 0.05 |

*The study period represents the days the patient remained mechanically ventilated up to a maximum of 14 from randomisation
